# Impact of COVID-19 Pandemic on Pathology Residency Program: An Experience from India

**DOI:** 10.5146/tjpath.2022.01587

**Published:** 2023-05-15

**Authors:** Arti Khatri, Somshankar Chowdhury, Dipti Sidam, Sonali Malik, Toshali Pandey, Sumanashree Mallappa

**Affiliations:** Department of Pathology, Chacha Nehru Bal Chikitsalaya, Delhi, India; ESI Medical College, Haryana, India; Gajra Raja Medical College, Gwalior, India; Maulana Azad Medical College, Delhi, India; Kodagu Institute of Medical Sciences, Karnatka, India

**Keywords:** Coronavirus, COVID-19, Pandemic, Pathology, Learning, Residency

## Abstract

***Objective:*** To study the impact of the COVID-19 pandemic on pathology residents through a questionnaire-based survey.

***Material and Method:*** We designed a Google survey questionnaire with 20-questions and distributed it to the pathology residents across India via e-mail and WhatsApp. All the responses collected were analysed using appropriate statistical methods.

***Results:*** We received a total of 81 responses. The majority (n=55, 68.8%) of the residents were aged 26-30 years with a male-female ratio of 1:2.2. Residents reported a significant decrease in classes as compared to pre-covid times. However, most institutions (90%) shifted to the virtual method for various teaching sessions. About 94.7% of the residents felt a fall in the quality of training due to Covid. A significant number of junior residents (76.92%) reported an inability to complete the target thesis enrolment. The residents saw a substantial decrease in the number of peripheral smears, bone marrow, cytology, and histopathology cases compared to pre-Covid times (p value <0.001 for all). An overwhelming 83.8% of the pathology residents were posted for COVID-19 duties. About 48.8% turned Covid positive. About 77.5% (n=62) of residents felt that the necessary training period would be extended.

***Conclusion:*** The COVID-19 pandemic has immensely affected the training and teaching of pathology residents in India. Similarly, this pandemic must have affected pathology residents all across the globe. Therefore, institutions can consider offering an extended period of up to one year, depending upon residents’ requests.

## INTRODUCTION

The coronavirus disease 2019 (COVID-19) pandemic has devastating effects on worldwide healthcare systems. Drastic measures had to be taken by various institutes universally to control its spread and impact. However, as the focus shifted primarily on the COVID-19 patient care and containment of the infection, the physical presence of students and staff was cut short in Pathology and other departments. These include cancellation of elective procedures, reduction in the volume of acute care surgery following surgical professional societies guidelines, and cancellation of lectures and offline conferences (1,2).

Education and training of the pathology residents while dealing with COVID-19 was a challenge in many institutions (3). Most institutions continued medical education (CME) programs and educational seminars by adhering to the strict social distancing recommendations. Some meetings have been postponed or held virtually, limiting their scope and outreach. In-person academic activities, including face-to-face teaching and simulation laboratories, have been interrupted. Many institutions resorted to innovative learning methods, digital pathology, teleconferencing, zoom meets, and online didactics to maintain the learning curves (1–3).

The pathology residents are affected by various factors like new learning modes, less on-site skills, and working away from the department. In addition, the emotional stress and breakdown on the news of illness and death of friends and family have caused challenges for many pathology training programs across the countries. It results in the disruption of existing training models for the residents. However, how much the teaching and training of pathology residents have been affected in India is unknown.

So far, only a very few studies have been done in India regarding the effect of this pandemic on residency programs. Till now, no such research has been published in the field of pathology. Therefore, in this study, we aimed to study the impact of the COVID-19 pandemic on pathology residents’ training programs in India.

## MATERIAL and METHOD

We designed a Google survey questionnaire with 20 questions to assess the different aspects of pathology teaching and learning. This study was conducted in compliance with the principles of the Declaration of Helsinki. The institute’s ethical committee approved the study protocol. The consent was obtained from the participants by attaching the consent form with the Google survey. The questionnaire covered various areas like haematopathology, cytopathology, and histopathology. We distributed it to both junior and senior residents of pathology all over India via WhatsApp (*Meta, USA*) and e-mail. Postgraduates or junior residents are students who have completed their graduation and have enrolled in a two- or three-year pathology residency programme to earn a postgraduate degree or a diploma respectively in the field of pathology. The senior residents are those who recently completed their pathology residency of two to three years. In India, senior residency is a personal decision that one can apply for one to three years. After completing the junior residency, one can also work as a consultant pathologist in any hospital or laboratory. The senior residents were also chosen for this study because they have experienced both pre-COVID and COVID-19 eras.

**We sent the questionnaire by e-mail and WhatsApp to the junior and senior residents of different institutes in India in the first week of July 2021. A time duration of 10 days was given to residents to fill up the survey.**

We collected the response data in Excel sheets.

Calculation of Sample Size: The sample size was calculated using Cochran’s formula.

N= z 2 p q/e 2.

Where

z =1.96

p = the (estimated) proportion of the population with the attribute in question.

q = 1 - p.

e = the desired level of precision (i.e., the margin of error),

Using the sample size of 4500 (derived from the National Medical Commission website, considering 1195 MD pathology seats every year, and one year of senior residency on an average. Senior residency is not compulsory in India, and not all opt for it after completing the post-graduation in pathology).

At a 90% confidence interval and margin of error of 9%, the population size is 82.

We did the statistical analysis using the Statistical Product and Service Solutions (SPSS) Statistics for Windows, Version 25.0 (Armonk, NY: IBM Corp). We used the Wilcoxon signed-rank test to analyse the differences between the pre-COVID-19 and COVID-19 responses. A p-value of less than 0.05 was taken as significant.

## RESULTS

**Figure 1:**
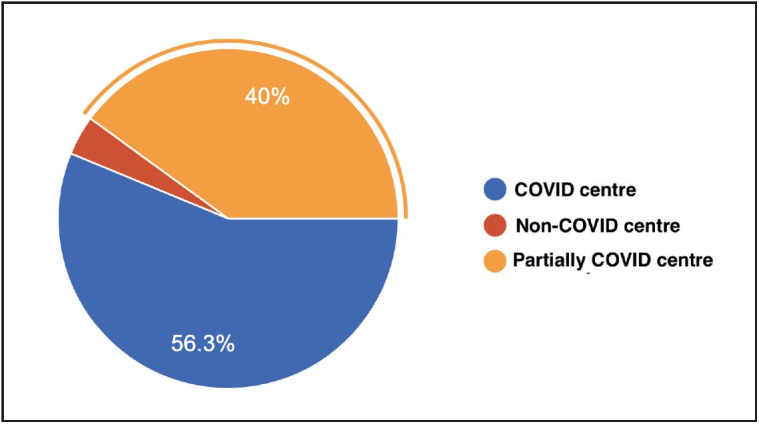
COVID- status of the institution as reported by respondents.

The questionnaire with responses is provided in Table 1. We received 81 responses from 22 institutions across 15 cities in India. One respondent refused to give consent, so we analysed 80 responses. The majority (n=55, 68.8%) of the respondents were females. Out of the respondents, 48.8 % (n=39) were junior residents and 51.2% (n=41) were senior residents. Most of the hospitals were functioning as COVID centres (56.3%), followed by partial COVID (40%) and non-COVID (3.7%) centres during the COVID-19 pandemic (Figure 1).

The residents reported a significant decrease in the clinicopathological case discussion (tumour board and clinicopathological meets) during the COVID-19 pandemic (Table 1).

**Table 1:** Questionnaire with responses

**S. No.**	**Question**	**Responses**				
1.	Institution status	Covid 56.3%		Non-Covid 3.7%	Partially Covid 40%	
2	Have you been posted in Covid duties?	Yes 83.8%			No 16.2%	
3	If yes to above, how many Covid duties have you performed so far?	<20 51.2%	20-50 17.5%	>50 13.7%	Not Applicable 17.5%	
4	Have you been Covid positive?	No 51.2%	Yes, mild symptoms 40%		Yes, was hospitalised 8.8%	
5	Have you ever had any apprehension regarding #	Catching Covid-19 60%	Transmitting to others 62.5%	No Apprehension 15%	Other apprehensions 5%	
6	What are your feelings during a pandemic? #	Anxious 36.3%	Depressed 42.5%	Burnt -out 25%	Fatigued 42.5%	No psychological issues 27.5%
7	Do you feel apprehension/ concerned about the fall in the quality of training due to Covid?	Yes 94.7%			No 5.3%	
8	How many classes/seminars have you attended per month?	Pre- Covid	<4 5%	4-8 40%	9-12 26.25%	>12 28.75%
		Covid	<4 80%	4-8 15%	9-12 1.25%	>12 3.75%
9	How many clinicopathological classes have you attended (per/year)?	Pre- Covid	<5 15.94%	5-10 28.98%	10-20 20.28%	>20 34.78%
		Covid	<5 80.26%	5-10 15.79%	10-20 2.63%	20 1.31%
10	Has your department shifted to virtual/online mode for classes	Yes 90%			No 10%	
11	Have you found these virtual/online classes useful	Yes 56.4%			No 43.6%	
12	Are you expecting to reach the target number of cases for thesis Completion? (Only Junior residents eligible to answer) n= 39.	Yes n= 9 23.08%		No n=30 76.92%	Not Applicable as I have already submitted thesis.	
13	If no, how much is the expected deficit for thesis cases enrolment?	<20% 16.66%	20-50% 53.33%	50-75% 23.33%	>75% 6.66%	
14	How many peripheral smears do you use to see during haematology posting per month?	Pre- Covid	<100 8.75%	100-500 25%	500-1000 31.25%	>1000 35%
		Covid	<100 61.25%	100-500 23.75%	500-1000 11.25%	>1000 3.75%
15	How many Bone marrow aspirates/biopsies did you use to see per month?	Pre- Covid	<5 20%	5-10 28.75%	10-20 27.5%	>20 23.75%
		Covid	<5 81.25%	5-10 12.5%	10-20 6.25%	>20 0
16	How many cytology cases did you use to see per month?	Pre- Covid	<50 11.25%	50-100 17.5%	100-200 31.25%	>200 40%
		Covid	<50 75%	50-100 15%	100-200 7.5%	>200 2.5%
17	How many biopsies/resection specimens do you use to see per month?	Pre- Covid	<50 7.5%	50-200 26.25%	200-400 26.25%	>400 40%
		Covid	<50 58.75%	50-200 30%	200-400 7.5%	>400 3.75%
18	Do you have any apprehension about the future because of lack of training due to the Covid-19 Pandemic?	Yes 87.5%			No 12.5%	
19	Do you think the training period needs to be extended to acquire adequate experience in pathology?	Yes 77.5%			No 22.5%	
20	If yes to the above, what is the appropriate period for the extension suitable for you?	3 months 12.9%	3-6 months 50%	6-12 months 29%	>12 months 8.1%	

# Participants could have multiple answers.

**Figure 2:**
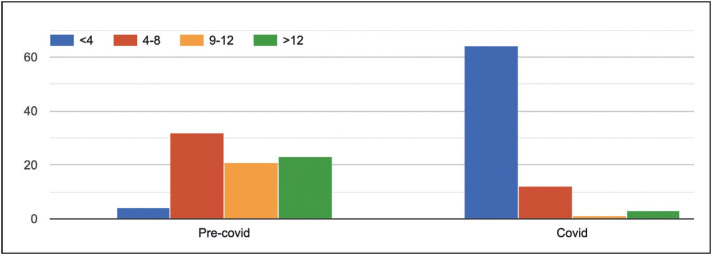
Number of classes attended per month in pre-COVID and post-COVID period.

There was a significant decrease in classes during the COVID-19 pandemic (p value<0.001) (Figure 2). In addition, the majority (n=72, 90%) of the residents reported that their institutions have shifted to a virtual teaching medium. However, only 55% (n=44) of the students found the online mode helpful.

Regarding thesis/ research work, only (n=9, 23.08%) residents could target thesis enrolment, while most (n=30, 76.92%) of the junior residents could not target thesis enrolment. Out of these junior residents who reported a deficit, 53.33% (n=16) and 23.33% (n=7) described a 20-50% and 50-75% deficit in the target number of cases required for their thesis completion, respectively.

There is a significant reduction in the number of cases seen by the residents in various areas of a pathology residency. In addition, there is a substantial reduction in the clinicopathological classes, number of peripheral smears, bone marrow smears, and biopsy/resection specimens examined by the residents compared to pre-covid times. Also, there is a significant reduction in the FNACs done and analysed. Approximately 95% (n=76) of the residents felt a fall in the quality of training due to the Covid pandemic.

**Figure 3:**
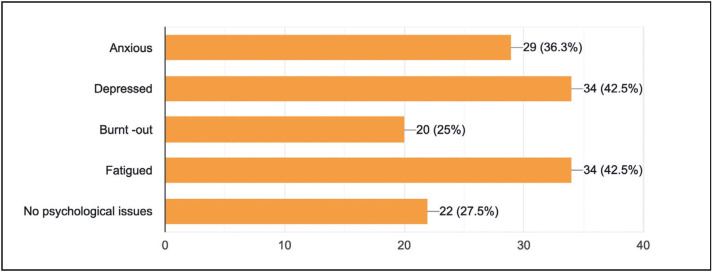
Feelings of residents during the COVID pandemic.

About 72.5% (n=58) of the residents suffered from various psychological issues like anxiety, depression, burn-out, fatigue, etc., while 27.5% (n=22) showed no psychological problems during this pandemic (Figure 3).

**Figure 4:**
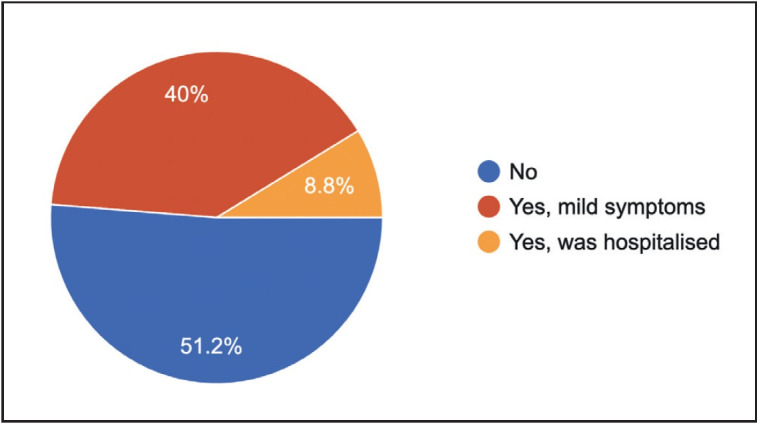
Status of COVID infection among residents.

An overwhelming 83.8% of the pathology residents were posted in Covid duties, and about 48.8% turned Covid positive (Figure 4).

About 77.5% (n=62) of residents felt that the training period needed to be extended by respective departments to acquire adequate experience in pathology. Out of the total residents who wanted an extension, approximately 12.9% (n=8), 50% (n=31), 29% (n=18), and 8.1% (n=5) of the residents thought that an extension of three months, three to six months, six months to one year, and more than one year was required to gain proper exposure for training in pathology, respectively.

## DISCUSSION

Pathology is a subject that can be mastered only by adequately grasping core concepts (2). To become a pathologist in India, one requires two to three years of vigorous training (junior residency) to achieve a Diploma and Degree in pathology. To achieve further experience in pathology training, a postgraduate degree holder can opt for a Senior residency program of up to three years in India. During residency, a pathology resident acquires skills like tissue processing, microscopic analysis, laboratory management, immunohistochemistry, haematopathology, cytopathology, transfusion medicine, etc. In the face of the ongoing COVID-19 pandemic, as the medical needs increase, so does the laboratory workload of the pathology department. Pathology residents worldwide have experienced various challenges in their training because of the pandemic.

Few articles reported the changes in the workflow in their institution amidst the Covid-19 pandemic. In their paper, Lieberman et al. (1) reported a significant disruption of daily medical student learning activities with the focus shifting to optimal patient care in their institution. They rapidly implemented substantial changes to medical education in clinical pathology to cope with it. They used distance learning platforms like teleconferencing, which is universally available to staff and students. Institutions also started with Covid -19 and distance learning classes to help the residents with the new platforms. They even had feedback scoring systems implemented at the end. Similarly, in their article on pathology residents, Cieri et al. (3) stated that to adhere to social distancing norms, the physical presence of staff and students in the pathology department was cut down. They reorganised the work logistics by introducing smart working, digital pathology, and checkpoint activities. Digital pathology was used for training across various disciplines of pathology. They converted glass slides into high-resolution data. Though they initially found it difficult to adjust to it away from the microscope, they later found it a much better alternative. They suggested the comprehensive implementation of digital pathology soon.

Furthermore, we found in this study that to manage the unprecedented need for an additional medical workforce, many residents from various specialties, including pathology personnel (around 84%), have been redeployed to cater to COVID-19 patients and wards, which causes significant stress to the resident doctors. These findings are similar to the study done by Romero et al. (4), and they reported that in the later part, as the Covid medical needs increased, first and second-year postgraduates were redeployed to work like interns and medical students. In contrast, third and fourth-year students were retained to handle pathology department workloads.

Romero et al. (4) also reported that educating pathology residents while maintaining COVID-19 norms was challenging. Resident rotations were rearranged with limited residents working on-site on rotation and others previewing the slides from residents’ rooms with a clear separation between residents working on-site and others. The transfusion medicine offered on-call phone assistance only. Daily check-in webinars and assignments were offered. Program directors periodically assessed residents for learning milestones. Leaving the laboratory to work for direct clinical care was stressful for many residents. Distance learning digital methods were introduced, and residents reported them helpful but also reported distractions during didactics. So, these methods though effective did not exactly replicate the pre-pandemic teaching and learning process.

In the light of these articles and the new hurdles faced by pathology residents, we highlighted the impact of the COVID-19 pandemic on pathology residents of India through a questionnaire-based survey.

### Use of smart methods, digital pathology, and virtual conferencing for teaching pathology

Virtual sessions have been adopted in various nations as a part of residency programs to compensate for the learning loss due to the pandemic. According to research by Hassell et al. (5), the constraints faced by pathology due to a pandemic necessitated modification to virtual means to avoid jeopardising teaching and training. This was an entirely new experience for the students, who had little or no access to hands-on instruction and relied entirely on a digital environment and virtual approaches to learn. For many, the abrupt move from traditional on-site and hands-on procedures to nearly virtual settings owing to a pandemic response has been unnerving. For example, a Pathology resident had to carry out case reviews, an essential component of training that shifted either entirely or partly to a virtual environment. The sudden switch to virtual teaching utilising digital technologies was laden with difficulties and an immediate perception that the teaching efficacy and quality had suffered (2,5).

There is a shift from hands-on learning to virtual mode. Although these options appeared effective, they still did not match the pre-pandemic offline teaching. However, some compensation has been offered to students by adopting virtual classes in various pathology departments. In India, as suggested by the current study, the majority of the pathology departments (90%) shifted to the online teaching mode. However, digital pathology in India is still developing, and not all are flexible in using the new methods.

### Redeployment of the residents to work in direct clinical care

Many of them were redeployed to the wards to treat Covid patients due to a shortage of clinicians. During this period, pathology residents have ably shared the burden of COVID-19 duties as around 83.8% of them have worked in Covid wards. Due to significant portions of time devoted to Covid patients and significantly less working time in various areas of pathology, academic teaching and research activities have suffered a lot. An article by Romero et al. (4) confirms the same.

### Covid-19 infection-related problems

Many residents have spent considerable time away from their training due to post-exposure self-isolation and hospital admissions while performing their front-line duties (6). In the current study, nearly 49% of the residents became Covid positive, and about 9% required hospitalisation for Covid-related complications.

### Emotional wellness

Not to be underestimated are the emotional and psychological effects of the pandemic. In addition to the constant stress of acquiring skills and providing care, the pressure to keep oneself healthy and prevent the virus’s community spread by not becoming a vector has been psychologically crippling for many doctors (6). About 75% of the residents faced various psychological issues in the current study. An article by Romero et al. (4) also talks about the emotional stress of keeping oneself safe and the news of losing near and dear ones, which crippled their residents.

Although virtual sessions are not entirely compensated for the offline class loss in pathology, virtual training is still beneficial in compensating for some losses, as shown by current study results. However, at the same time, some specialities like surgical branches bear the most burnt as virtual learning is highly restricted, as evidenced by the fact that during the COVID-19 epidemic, the majority of residents reported not receiving any educational sessions from the university or surgery department staff during Covid-19 pandemic (7).

Hence from this study, we found out that pathology training in India has suffered during the COVID-19 pandemic. Also, residents in the different pathology subspecialties saw a significant caseload reduction compared to pre-Covid times. Similar to this study, Hassell et al. reported that most professors and program directors believed that the transition had affected the quality (59% and 62%, respectively) and efficacy (66%) of teaching pathology. Similarly, residents shared this opinion regarding the adverse impact on quality (59%) and effectiveness (64%) of learning pathology (5).

Most of the residents are stressed and concerned about the quality of their training. Therefore, most felt their training period should be extended to compensate for the lost time.

It thus has become necessary to balance the social distancing norms needed to slow the disease spread while ensuring that trainee doctors’ educational and clinical needs are minimally affected. Similarly, doctors in their training period today shall soon manage laboratories or do the reporting independently concerning the pathology training (2).

Based on the findings, we recommend that the institutes consider offering an extension of up to one year to the pathology residents all across India. Furthermore, as per the study by Azimi Khatibani et al. (2), investment in virtual telepathology infrastructure and training all the staff and residents to use the same is a need of the hour.

The limitation of this study is that not all the residents have responded to the survey, but we still have a large enough sample to represent a pan-India scenario. The strength of this study is that all the aspects of pathology residency, research, and academics have been included. We have also incorporated the residents’ feelings about their training and provided a possible solution.

## CONCLUSION

The COVID-19 pandemic has adversely affected the pathology residents all across India. Similarly, this pandemic must have affected pathology residents all across the globe. However, the exact impact is still unknown. It is difficult to predict how long this pandemic will last, and the pathology fellows and residents need to continue their learning and training. Whatever loss has happened to them due to the COVID -19 pandemic so far has to be compensated by the institutions by giving them the option of extension of their training period to offset this significant loss by the residents. We hope our results help better the residency training programs, understand the pandemic’s influence, and modify pathology teaching program durations accordingly to provide more effective training.

## Conflict of Interest

We did not receive payment or services from a third party (government, commercial, private foundation, etc.) for any aspect of the submitted work. We have no financial relationship with any third party(government, commercial, private foundation, etc.).

There are no relationships/conditions/circumstances related to this work that present a potential conflict of interest.
